# Supplementary material for: Water-Soluble Pristine C60 Fullerene Inhibits Liver Alterations Associated with Hepatocellular Carcinoma in Rat
Source: Pharmaceutics. 2020 Aug 22;12(9):794. doi: 10.3390/pharmaceutics12090794 (PMC7559840; doi:10.3390/pharmaceutics12090794)
Supplement: Supplementary file 1 [file pharmaceutics-12-00794-s001.pdf]

pharmaceutics

Supplementary Materials: Water-Soluble Pristine C_{60} Fullerene Inhibits Liver Alterations Associated with Hepatocellular Carcinoma in Rat

Halyna Kuznietsova , Natalia Dziubenko , Tetiana Herheliuk [1], Yuriy Prylutskyy ,
Eric Tauscher [2], Uwe Ritter and Peter Scharff

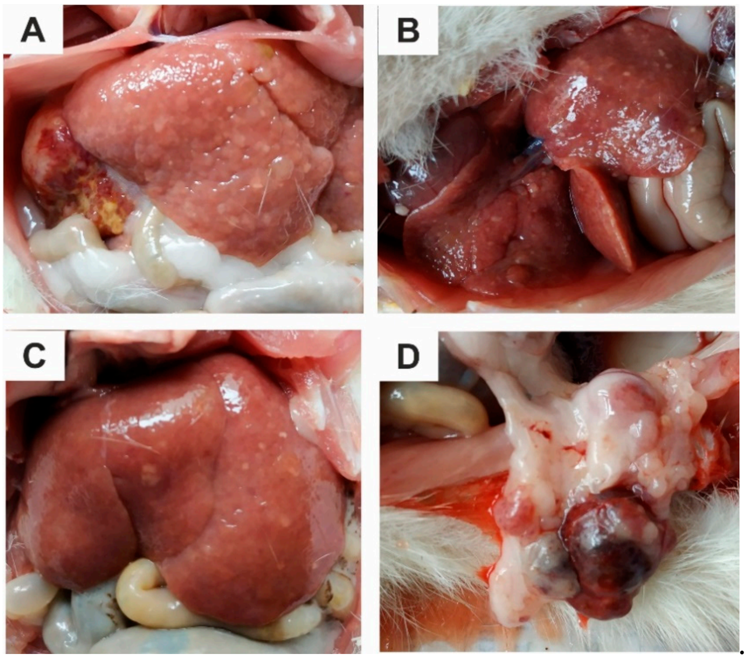

Figure S1. Representative rat livers after 54 weeks from the start of the experiment. (**A**): HCC; (**B**): HCC + 5FU; (**C**) HCC + C_{60}FAS; (**D**): pancreatic metastasis (group 5FU).

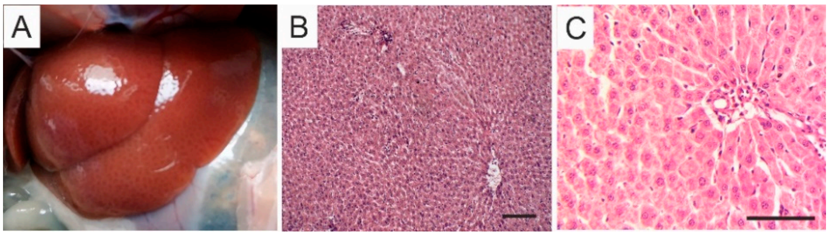

Figure S2. Representative liver of healthy rats received C_{60}FAS at the time of the sacrifice (**A**) and H&E staining of liver tissue, magnification ×100 (**B**), scale 200 µm, and ×400 (**C**), scale 100 µm.P

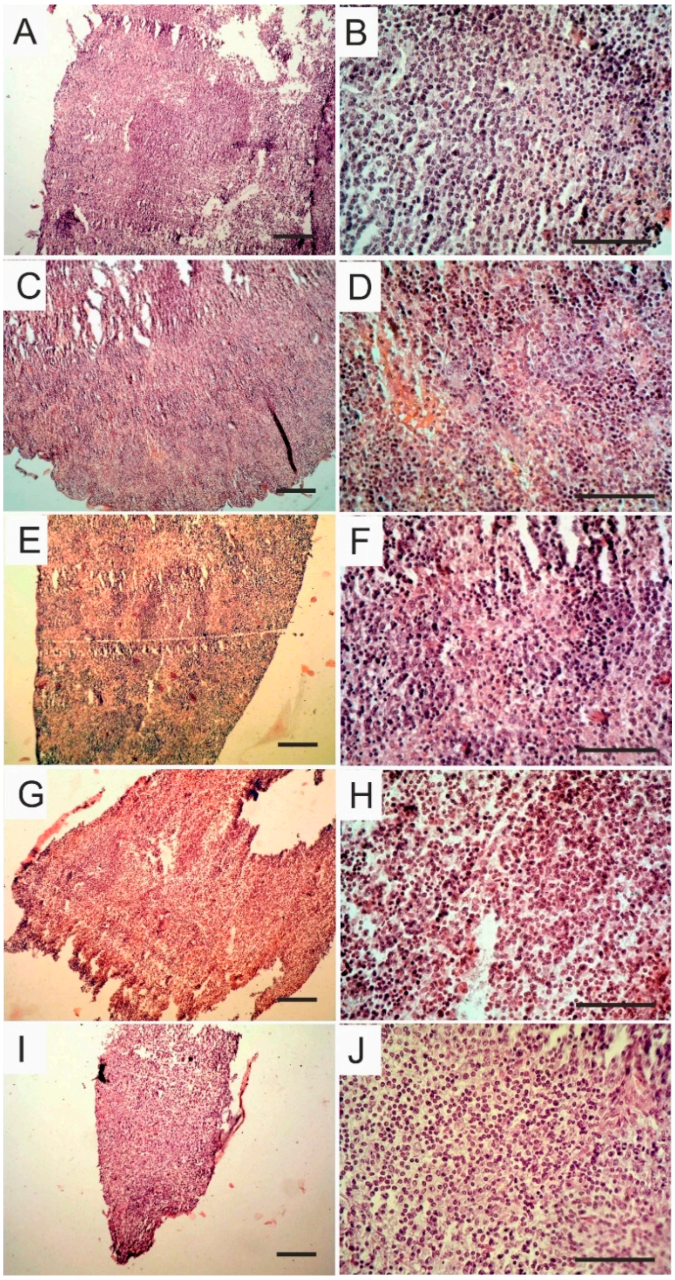

Figure S3. Microphotographs of rat spleen, H&E staining, magnification ×100 (**A**,**C**,**E**,**G**,**I**), scale 200 μm, and ×400 (**B**,**D**,**F**,**H**,**J**), scale 100 μm: (**A**,**B**)—control; (**C**,**D**)—C_{60}FAS; (**E**,**F**)—HCC; (**G**,**H**)—HCC + 5FU; (**I**,**J**)—HCC + C_{60}FAS.

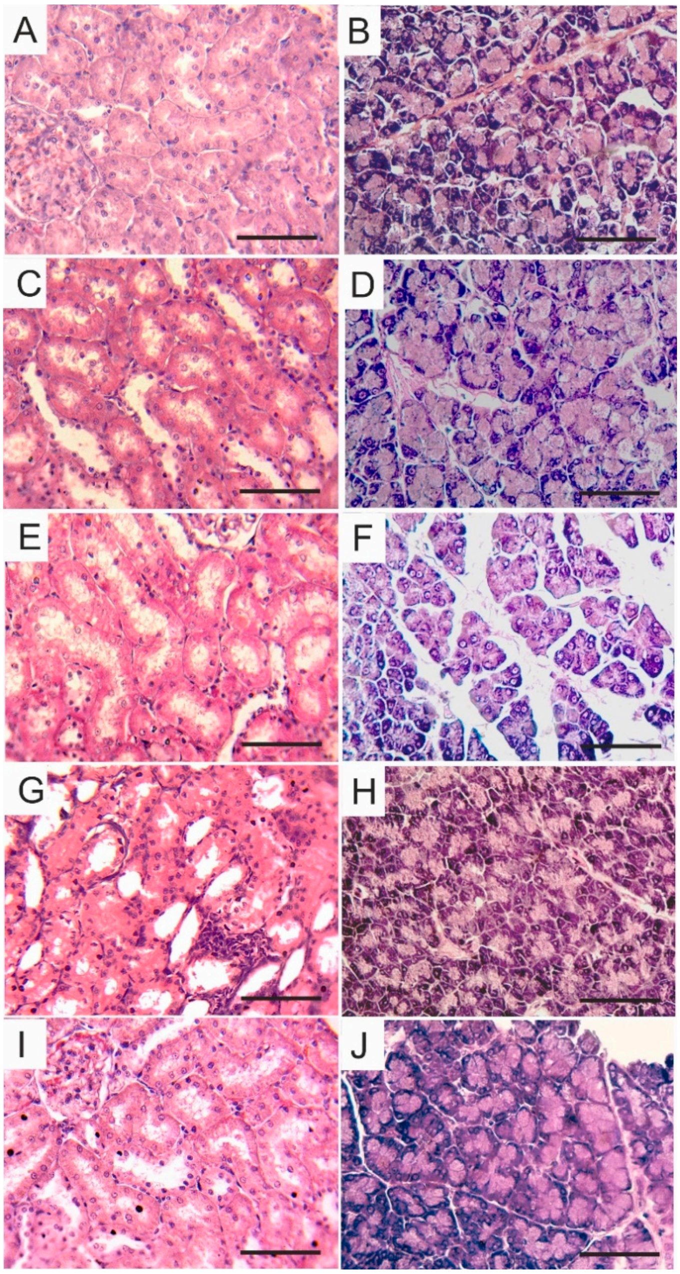

Figure S4. Microphotographs of rat kidney (**A**,**C**,**E**,**G**,**I**) and pancreas (**B**,**D**,**F**,**H**,**J**), H&E staining, magnification ×400, scale 100 μm: (**A**,**B**)—control; (**C**,**D**)—C_{60}FAS; (**E**,**F**)—HCC; (**G**,**H**)—HCC + 5FU; (**I**,**J**)—HCC + C_{60}FAS.
